# Mechanisms involved in xyloglucan catabolism by the cellulosome-producing bacterium *Ruminiclostridium cellulolyticum*

**DOI:** 10.1038/srep22770

**Published:** 2016-03-07

**Authors:** Julie Ravachol, Pascale de Philip, Romain Borne, Pascal Mansuelle, María J. Maté, Stéphanie Perret, Henri-Pierre Fierobe

**Affiliations:** 1 Aix-Marseille Université-CNRS, Laboratoire de Chimie Bactérienne, UMR7283, IMM, 31 chemin Joseph Aiguier, F-13402 Marseille, France; 2 Plate-forme de Protéomique, IMM, 31 chemin Joseph Aiguier, F-13402 Marseille, France; 3 Aix-Marseille Université-CNRS, Laboratoire d’Architecture et Fonction des Macromolécules Biologiques, UMR7257, Parc Technologique de Luminy, F-13288 Marseille, France

## Abstract

Xyloglucan, a ubiquitous highly branched plant polysaccharide, was found to be rapidly degraded and metabolized by the cellulosome-producing bacterium *Ruminiclostridium cellulolyticum*. Our study shows that at least four cellulosomal enzymes displaying either endo- or exoxyloglucanase activities, achieve the extracellular degradation of xyloglucan into 4-glucosyl backbone xyloglucan oligosaccharides. The released oligosaccharides (composed of up to 9 monosaccharides) are subsequently imported by a highly specific ATP-binding cassette transporter (ABC-transporter), the expression of the corresponding genes being strongly induced by xyloglucan. This polysaccharide also triggers the synthesis of cytoplasmic β-galactosidase, α-xylosidase, and β-glucosidase that act sequentially to convert the imported oligosaccharides into galactose, xylose, glucose and unexpectedly cellobiose. Thus *R. cellulolyticum* has developed an energy-saving strategy to metabolize this hemicellulosic polysaccharide that relies on the action of the extracellular cellulosomes, a highly specialized ABC-transporter, and cytoplasmic enzymes acting in a specific order. This strategy appears to be widespread among cellulosome-producing mesophilic bacteria which display highly similar gene clusters encoding the cytosolic enzymes and the ABC-transporter.

Plant cell walls are essentially composed of an intricate network of polysaccharides and lignin. The most abundant polysaccharide, the cellulose, is a highly homogenous linear polymer exclusively composed of β-1,4 linked glucosyl residues forming crystalline microfibrils. The latter are surrounded by hemicellulose and pectin, and also lignin in secondary plant cell walls. The terms hemicellulose and pectin designate highly heterogeneous groups of branched polysaccharides, whose degree of polymerization, main chain, decoration, and solubility are particularly variable. One of the most abundant hemicellulosic polymers is xylan, whose backbone is composed of β-1,4 linked xylosyl residues, which can display a number of side chains which vary tremendously among plants, or among tissues within the same plant^1^. Xyloglucan is another prominent hemicellulosic polysaccharide, especially in primary cell walls^2^,^3^, where it interacts with the cellulose microfibrils. The backbone of xyloglucan is similar to a cellulose chain, with β-1,4 linked glucosyl residues that carry side chains. The decorations displayed by xyloglucan can also vary but one of the most prominent motifs is a repeated sequence “XXXG”^4^ of three glucosyl residues substituted with a single α-1,6 xylosyl (X), followed by an unbranched glucosyl residue (G). Quite often the second and/or third xylosyl residue can be substituted with a β-1,2 galactosyl residue (L), as observed in tamarind xyloglucan (Fig. 1), which can be further decorated with an α-1,2 fucosyl residue, as found in *Arabidopsis taliana*^5^.

The total saccharification of plant cell walls thus requires a vast arsenal of enzymes displaying various activities and specificities, which is rarely met in a single microorganism. The largest collections of plant cell wall degrading enzymes are frequently observed in cellulolytic organisms. These microorganisms usually synthesize an array of cellulases that depolymerize cellulose, but are also often well equipped for hemicellulose and pectin degradation as they produce a set of enzymes which collectively hydrolyze to various extents the other polysaccharides surrounding the cellulose microfibrils.

In aerobic biotopes, the cellulolytic microorganisms usually secrete copious amounts of free cellulases and hemicellulases in the environment. In contrast, most anaerobic cellulolytic bacteria gather the plant cell wall degrading enzymes in million-Da range extracellular complexes termed cellulosomes^6^,^7^,^8^,^9^, sparingly produced compared to aerobic microorganisms. The simplest cellulosomes such as those produced by the model bacterium *Ruminiclostridium cellulolyticum* (formerly known as *Clostridium cellulolyticum*^10^) are composed of a single primary scaffoldin which binds to crystalline cellulose *via* a family-3a Carbohydrate Binding Module (CBM)^11^. The scaffoldin also contains eight receptor domains called the cohesins which interact tightly with a complementary module borne by the catalytic subunits and called the dockerin. In the case of *R. cellulolyticum*, the cohesin/dockerin system is non-specific, *i.e.* any enzyme dockerin can bind to any of the 8 cohesins displayed by the scaffoldin with similar affinity^12^,^13^. In contrast, the cellulosomes synthesized by other anaerobic bacteria, such as *R. thermocellum* (formerly known as *Clostridium thermocellum*^10^) or *Ruminococcus flavefaciens*, can be much more complex and include several interacting scaffoldins, as well as several types of specific cohesin/dockerin docking devices^9^.

Most of the cellulases produced by *R. cellulolyticum* have been previously characterized in the free and complexed states^14^,^15^,^16^,^17^,^18^,^19^,^20^,^21^ but the genome of *R. cellulolyticum* also displays many genes that encode putative Glycoside Hydrolases (GH) predicted to target both the backbone and the side chains of various hemicellulosic polysaccharides^22^,^23^. Furthermore, *R. cellulolyticum* is capable to metabolize some of these polysaccharides like xylan^23^, in contrast to *R. thermocellum* for which the hydrolysis of xylan is “only” expected to make the cellulose microfibrils accessible to the cellulosomes^24^.

Recently, among the cellulosomal family-9 Glycoside Hydrolases (GH9) of *R. cellulolyticum*, Cel9X, predicted to be a cellulase, was described as highly active only on xyloglucan^19^, and the enzyme Cel9U, whose preferred substrate is cellulose, was found to also display an elevated activity on xyloglucan^19^. These observations raised the question whether the bacterium can metabolize this highly branched substrate. To date, xyloglucan utilization was described for only two Gram-negative bacteria, a human gut *Bacteroidetes*^25^ and the saprophyte *Cellvibrio japonicus*^26^. A similar model of xyloglucan utilization was proposed for both microorganisms including an external degradation of xyloglucan by outer membrane/secreted xyloglucanases, followed by the import of produced short oligosaccharides *via* a TonΒ-dependent sugar receptor/transporter in the periplasm, where their subsequent hydrolysis into monosaccharides occurs.

To our knowledge, the present report is the first investigation of the mechanisms involved in xyloglucan utilization by a Gram-positive bacterium. Our study revealed that *R. cellulolyticum* rapidly grows on xyloglucan and developed a quite different strategy to metabolize this polysaccharide. Our investigations were conducted at several levels: we first identified and characterized the major cellulosomal enzymes involved in the extracellular degradation of xyloglucan. In a second step, we established the role of a highly specific ATP-binding cassette transporter in the import of xyloglucan oligosaccharides produced by the cellulosomal xyloglucanases. Finally, we identified three cytoplasmic enzymes acting in a specific order to convert the imported xyloglucan oligosaccharides into the assimilable simple sugars glucose, xylose and galactose but also unexpectedly the disaccharide cellobiose. On the basis of all these data, a general model for xyloglucan utilization by *R. cellulolyticum* and other cellulosome-producing bacteria is proposed.

## Results

### Growth of *R. cellulolyticum* on xyloglucan and xyloglucan-derived oligosaccharides

The unexpected high activity formerly reported for the cellulosomal enzymes Cel9X and Cel9U on xyloglucan^19^ suggested that *R. cellulolyticum* may have the capacity to metabolize this branched polysaccharide. To assess this hypothesis, the strain was cultivated on minimal medium containing xyloglucan at 3.5 g/L as the sole carbon source (supplemental Fig. S1a). *R. cellulolyticum* was found to grow rapidly on the branched substrate, displaying a rather short doubling time (10 ± 0.7 h) during the exponential phase of growth, in a range similar to that reported for cultivation on cellobiose-containing minimal medium (7 ± 0.8 h)^27^,^28^.

**Figure 1:**
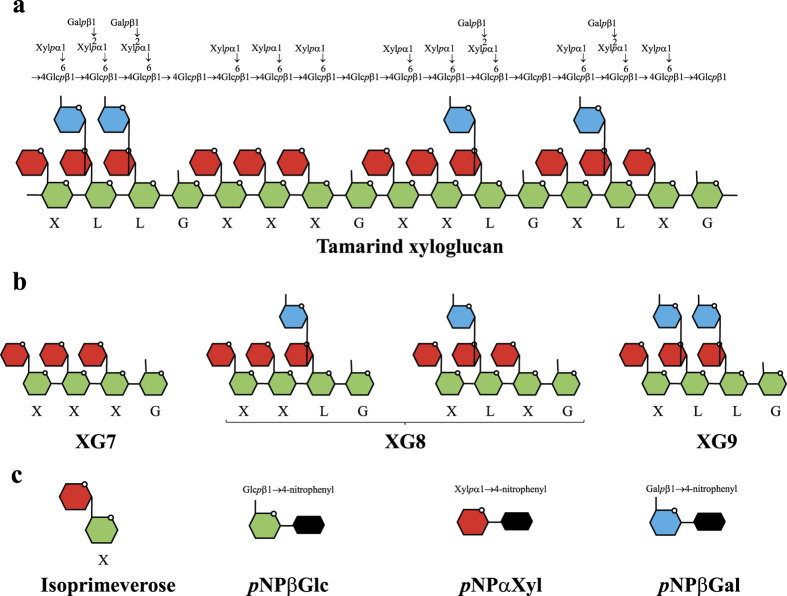
Schematic representation of the substrates used. (**a**) Two representations of the general structure of tamarind xyloglucan. (**b**) Four-glucosyl xyloglucan oligosaccharides (XGO_4_). (**c**) Isoprimeverose and aryl glycosides. Xyloglucan portions are named according to reference^4^.

To further investigate the growth capacities of *R. cellulolyticum*, 3.5 g/L of xyloglucan were hydrolyzed *in vitro* using a high concentration of the xyloglucanase Cel9X for 24 hours. The polysaccharide was completely converted into a mixture of 4-glucosyl xyloglucan oligosaccharides (XGO_4_) XXXG, XLXG, XXLG and XLLG (Fig. 1) as shown by analysis using High Pressure Anion Exchange Chromatography coupled with Pulsed Amperometric Detection (HPAEC-PAD) (supplemental Fig. S2). The obtained mixture was used to prepare a minimal medium containing the XGO_4_ at a final concentration of 3.5 g/L as the sole carbon source. *R. cellulolyticum* was found to grow on xyloglucan oligosaccharides (supplemental Fig. S1b) but displayed a slightly extended doubling time (17 ± 0.4 h) compared to that determined on xyloglucan-based medium, that might be due to a carbon overflow of the glycolytic pathway^29^,^30^. Nevertheless, these experiments clearly showed that *R. cellulolyticum* can metabolize both xyloglucan and XGO_4_.

### Extracellular degradation of xyloglucan by the cellulosomes and purified cellulosomal enzymes

**Figure 2:**
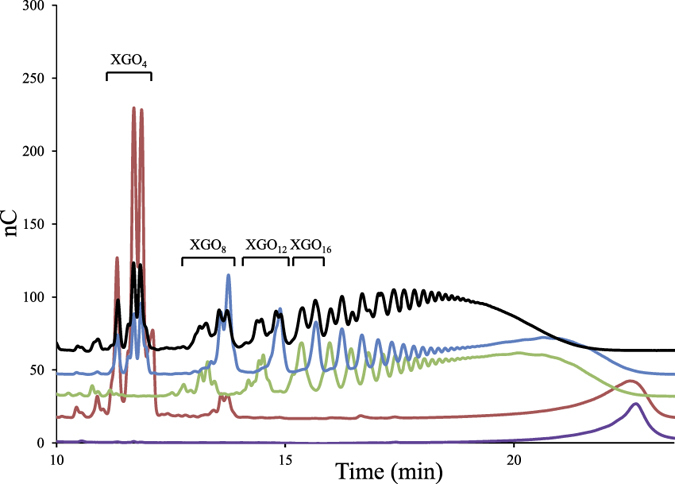
Xyloglucan degradation patterns by purified cellulosomes, Xgh74A, Cel44O, or Cel9X. The samples were analyzed by HPAEC-PAD. Xyloglucan at 3.5 g/L was incubated for 3 h at 37 °C with no enzyme (purple), with 6 mg/L (approx. 10 nM) of purified cellulosomes (black), with 2.5 nM of Xgh74A (red), with 2.5 nM of Cel44O (green), or with 2.5 nM of Cel9X (blue). “XGO_4_”, “XGO_8_”… “XGO_16_” refer to xyloglucan oligosaccharides displaying 4, 8…16 glucosyl residues backbone, respectively.

The hydrolytic activity of the purified cellulosomes was assayed at approx. 10 nM (6 mg/L) on 3.5 g/L xyloglucan. The cellulosomes, known to contain Cel9X and Cel9U^22^, were found to rapidly depolymerize the branched polysaccharide, with a specific activity estimated at 547 ± 8.4 IU/μM. HPAEC-PAD analyses revealed that during the kinetic (Fig. 2), the cellulosomes produced xyloglucan oligosaccharides of various lengths, part of which are superimposed with those generated by the cellulosomal xyloglucanase Cel9X, or the cellulase Cel9U which releases the same oligosaccharides as Cel9X^19^. Interestingly the cellulolytic complexes also produced other oligosaccharides that neither Cel9X nor Cel9U can generate, thereby suggesting the cellulosomes contain other enzyme(s) acting differently on the highly decorated polysaccharide (Fig. 2). The analysis of the carbohydrate active enzymes (CAZymes^31^) content encoded by the genome of *R. cellulolyticum* suggested that two other cellulosomal enzymes might exhibit some activity on xyloglucan^22^.

**Figure 3:**
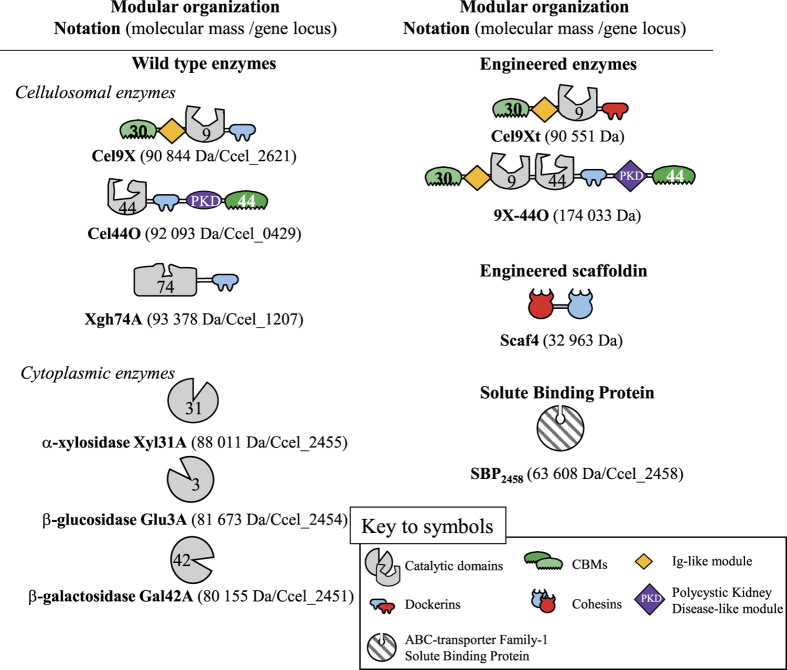
Schematic representation of the recombinant proteins used in this study. The GH- and CBM-families are indicated. Blue cohesins and dockerins designate interacting modules from *R. cellulolyticum*; red cohesins and dockerins designate interacting modules from *R. thermocellum*. Cel9Xt designates Cel9X from *R. cellulolyticum* bearing a *R. thermocellum* dockerin. 9X-44O designates an engineered fusion enzyme encompassing Cel9X (without the C-terminal dockerin) as the N-terminal moiety and Cel44O as the C-terminal moiety. The loci of the genes are given according to NCBI (http://www.ncbi.nlm.nih.gov/nuccore/NC_011898).

The most evident candidate was the putative enzyme Xgh74A, encoded by the gene at locus Ccel_1207, and composed of a GH74 catalytic module and a C-terminal dockerin (Fig. 3). Most of the fifteen GH74 characterized to date were described as xyloglucanases, suggesting Xgh74A might also degrade this polysaccharide^31^. The mature form of the protein was overproduced in *Escherichia coli* and purified. The recombinant enzyme displayed a high activity on xyloglucan with *k*_*cat*_ and *K*_m_ values of 2,304 min^−1^ and 0.37 g/L, respectively, thus leading to a *k*_*cat*_/*K*_m_ eight times higher than that of Cel9X (supplemental Table S1). The HPAEC-PAD analysis of the activity of Xgh74A on xyloglucan (Fig. 2) shows that the enzyme cleaves the same glycosidic linkage of the backbone as Cel9X (XXXG↓XXX). Nevertheless Xgh74A is clearly an exoxyloglucanase (Fig. 2), which almost exclusively released XGO_4_ (XXXG, XLXG, XXLG and XLLG) and whose activity did not significantly reduce the viscosity of the substrate solution (supplemental Fig. S3), whereas Cel9X unambiguously displays an endo mode of action (Fig. 2 and S3). Sequence comparison with other GH74 enzymes indicates that Xgh74A displays both the active-site blocking extra loop (N387-K398) responsible for exo-activity in another GH74 xyloglucanase^32^,^33^, and also the active site tryptophan pair (W341-W342) known to be essential for processive degradation by an endo-processive xyloglucanase^34^, thereby suggesting Xgh74A might act as an exo-processive xyloglucanase. Xgh74A also exhibited significant activities on the soluble cellulose CarboxyMethyl Cellulose (CMC) and amorphous cellulose (PASC) in contrast to Cel9X whose activity is restricted to xyloglucan (Table 1).

The second selected enzyme that might be active on xyloglucan was Cel44O, encoded by the gene at locus Ccel_0429. The modular organization of Cel44O includes an N-terminal GH44 catalytic module followed by a dockerin module, a Polycystic Kidney Disease (PKD)-like domain and a C-terminal family 44 CBM (Fig. 3). Most of the fifteen characterized GH44 enzymes were described as cellulases^31^, and consequently Cel44O was formerly predicted to be a cellulose-hydrolyzing enzyme^22^. Nevertheless, at least five bacterial GH44 enzymes were reported to display elevated xyloglucanase activities, including the C-terminal moiety of the bifunctional enzyme CelJ from *R. thermocellum*^35^. This truncated form of the enzyme, termed Cel44A^35^, which exhibits comparable activities on xyloglucan and CMC, is highly similar to Cel44O (59% sequence identity) and shares the same modular organization (GH44-dockerin-PKD-CBM44) as Cel44O^19^ (Fig. 3). Thus, the uncertainty regarding the putative activities of Cel44O prompted us to overproduce this enzyme in *E. coli* for subsequent purification and characterization. Interestingly, the N-terminal part of CelJ^36^ called Cel9D is highly similar (53% and 68% of sequence identity and similarity, respectively) to Cel9X from *R. cellulolyticum* exhibiting the same overall domain arrangement (CBM30-Ig-GH9)^19^, though Cel9D was described as a cellulase^36^. Nevertheless, this observation suggests that Cel9X and Cel44O from *R. cellulolyticum* and CelJ from *R. thermocellum* are phylogenetically related, and incited us to merge Cel9X and Cel44O to create a novel bi-functional enzyme called 9X-44O (Fig. 3) mimicking CelJ. This protein was also purified from an *E. coli* overproducing strain.

**Figure 4:**
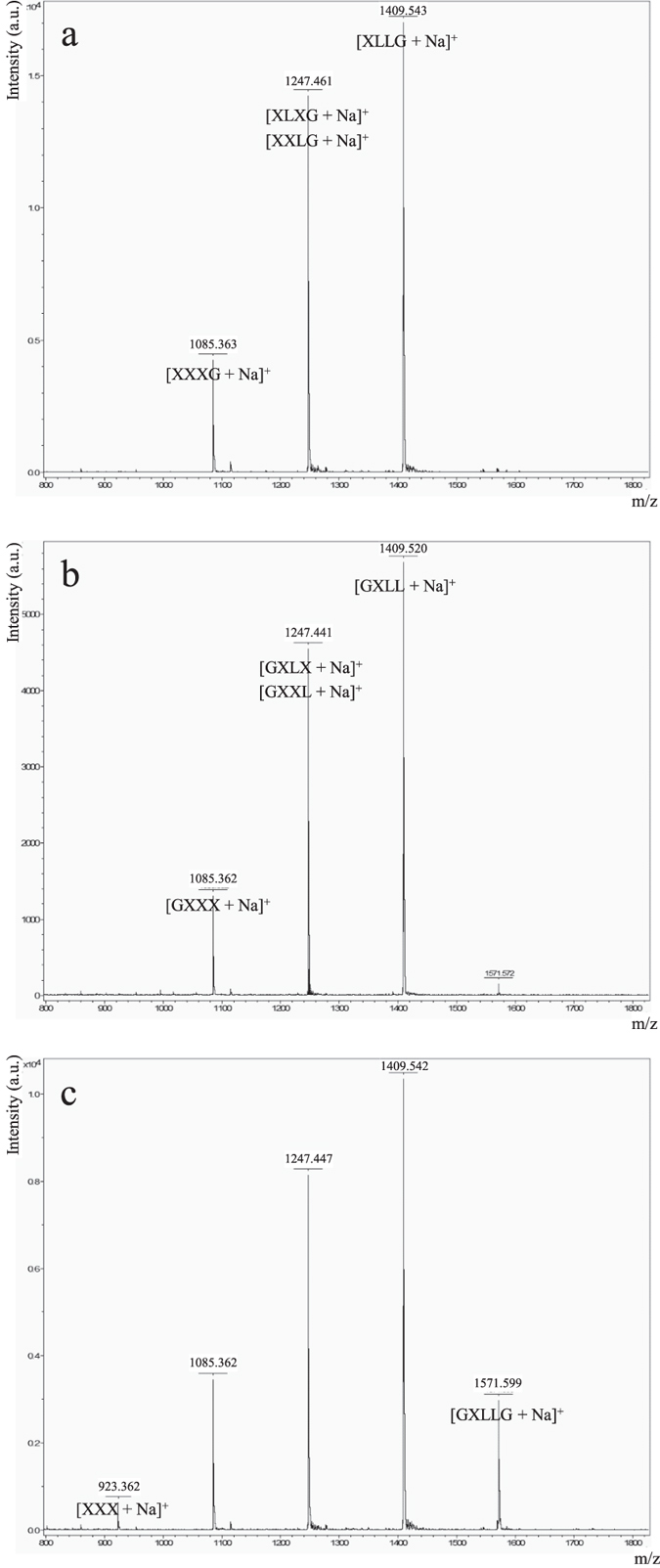
Mass spectrometry analysis of the end products of Cel9X, Cel44O and 9X-44O on xyloglucan. Xyloglucan (3.5 g/L) was incubated for 24 h at 37 °C with 0.1 μM of Cel9X (**a**), 0.2 μM of Cel44O (**b**), and 0.1 μM of 9X-44O (**c**). The complete degradation of the polysaccharide was verified in each case by HPAEC-PAD. One μL was then mixed with 50% CH_3_CN in water (v/v) supplemented with 0.1% (v/v) folic acid. The molecular masses (in Da) are indicated on each peak as well as the composition of the corresponding oligosaccharide(s) except for (**c**) where the composition is only specified for the peaks displaying the lightest and heaviest masses. u. a., arbitrary unit.

The characterization of Cel44O identified this enzyme as an endocellulase displaying elevated activities on CMC and amorphous cellulose. As shown in Table 1, grafting Cel9X at the N-terminus of Cel44O in the bi-functional enzyme 9X-44O did not alter the activities of Cel44O on cellulosic substrates, which remained in the same range. Nevertheless, though CMC is its favorite substrate, Cel44O also exhibited an important activity on xyloglucan (Table 1). Interestingly, on the latter substrate, HPAEC-PAD analyses showed that different oligosaccharides were released by Cel44O (Fig. 2) compared to those produced by Cel9X, Cel9U and Xgh74A. Furthermore, their retention times coincided with the unidentified oligosaccharides produced by the cellulosomes. Examination of the end products generated by Cel44O and Cel9X using mass spectrometry (Fig. 4) provided similar molecular masses in both cases: 1,085.4, 1,247.5 and 1,409.5 Da, thereby indicating that the oligosaccharides released by Cel9X and Cel44O most probably display the same glycosidic compositions. The same masses were also obtained for the end products generated by the bi-functional enzyme 9X-44O, but two additional peaks of molecular masses 923.4 (corresponding to 3 xylosyl and 3 glucosyl residues, XXX) and 1,571.6 (corresponding to 2 galactosyl, 3 xylosyl and 5 glucosyl residues, GXLLG) were also observed. Altogether these data strongly suggested that Cel44O, as formerly reported for another GH44 xyloglucanase^37^, cleaves the main chain as follows: XXX↓GXXX. This assumption was further verified using a specific β-glucosidase Glu3A (see below and Fig. 3) that specifically hydrolyses undecorated glucosyl residues at the non-reducing end of xyloglucan oligosaccharides (supplemental Fig. S4), thereby enabling us to propose the following XGO_4_ mixture GXXX, GXLX, GXXL and GXLL as the end products generated by Cel44O on tamarind xyloglucan (Fig. 4).

**Figure 5:**
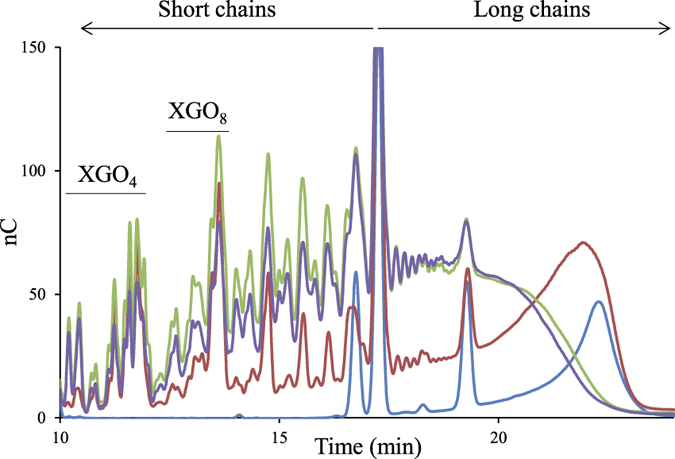
Xyloglucan degradation patterns by the free enzyme pair Cel44O + Cel9Xt, 9X-44O and the minicellulosome Scaf4(Cel9Xt + Cel44O). The samples were analyzed by HPAEC-PAD. Xyloglucan (at 3.5 g/L) was incubated for 24 h at 37 °C without enzyme (light blue) or with 1 nM of Cel9Xt and 1 nM Cel44O (red), 1 nM of 9X-44O (green) or 1 nM of the complex Scaf4(Cel9Xt + Cel44O) (dark blue). “XGO_4_” and “XGO_8_” refer to xyloglucan oligosaccharides displaying 4 and 8 glucosyl residues backbone, respectively.

As shown in Table 1, the bi-functional 9X-44O exhibited a specific activity on xyloglucan (based on the initial velocity) similar to that of Cel9X and Cel44O, or an equimolar mixture of free Cel44O and an engineered form of Cel9X whose native C-terminal dockerin was replaced by a *R. thermocellum* dockerin (Cel9Xt, Fig. 3). Nevertheless, longer incubation times revealed an improved depolymerization of xyloglucan compared to the free enzyme mixture (Fig. 5), leading to reduced proportions of long chains, whereas larger amounts of short oligosaccharides were produced. Interestingly, a similar degradation pattern was also obtained for the minicellulosome containing Cel9Xt and Cel44O bound onto the hybrid scaffoldin Scaf4^19^ (Fig. 3) harboring one cohesin from *R. cellulolyticum* and one cohesin from *R. thermocellum*. Thus, gathering Cel9X and Cel44O either by complexation onto a scaffoldin or by a covalent linkage improved their capacity to depolymerize xyloglucan.

### Expression profile of the genes putatively involved in xyloglucan catabolism

**Figure 6:**
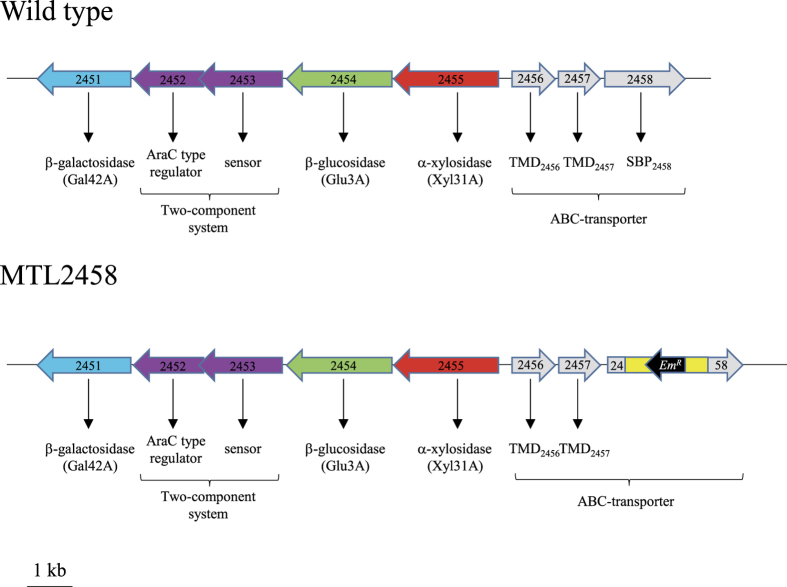
Schematic representation of the clusters putatively involved in the import and metabolism of xyloglucan oligosaccharides in wild-type and mutant MTL2458 strains of *R. cellulolyticum*. Genes and their orientation are given as arrows. The “Ccel” loci numbers (given according to NCBI http://www.ncbi.nlm.nih.gov/nuccore/NC_011898) are indicated in each gene, and the predicted function of the corresponding proteins are shown at the bottom of each gene. TMD designate the putative TransMembrane Domains and SBP the putative Solute Binding Protein. Em^R^ designates the erythromycin-resistance cassette and the yellow box represents the *Lactococcus lactis* type II intron^41^ in the mutant strain MTL2458.

None of the cellulosomal enzymes (Cel9X, Cel9U, Cel44O and Xgh74A) displaying an elevated activity on xyloglucan, nor the purified cellulosomes were found to hydrolyze the xylogucan oligosaccharides XXXG, XLXG XXLG or XLLG, the degradation of which into fermentable monosaccharides requires additional enzymatic activities, *i.e.* α-xylosidase, β-glucosidase and β-galactosidase activities. The genome of *R. cellulolyticum* displays three genes that putatively encode a GH31 α-xylosidase (Xyl31A), a GH3 β-glucosidase (Glu3A) and a GH42 β-galactosidase (Gal42A) at loci Ccel_2455, Cel_2454 and Ccel_2451, respectively^22^. These genes are located in a cluster (Fig. 6) containing also two genes predicted to encode a two-component system^38^ comprising a sensor (locus Ccel_2453) and an AraC-like transcriptional regulator (locus Ccel_2452). The three putative enzymes encoded by this gene cluster lack typical signal sequences, suggesting that XGO_4_ have to be imported in the cytoplasm for subsequent degradation into fermentable sugars. Interestingly, a divergent cluster of genes located immediately downstream of the 5-gene cluster putatively encodes an ATP-binding cassette transporter (ABC-transporter) predicted to transport sugar encompassing two TransMembrane Domains (TMD_2456_ and TMD_2457_ encoded by genes at loci Ccel_2456 and Ccel_2457, respectively) likely to constitute a heterodimer transmembrane channel, and a family 1 extracellular Solute-Binding Protein (SBP_2458_) containing a type II leader peptide (locus Ccel_2458). No ATP-binding protein encoding gene could be found within the divergent clusters or the surrounding DNA regions, but quite often in Gram-positive bacteria the gene coding for the ATP-binding protein is not part of a specific transporter operon and can function with several different transporters^39^,^40^. The proximity of the genes coding for the cytoplasmic enzymes and the ABC-transporter suggested the latter might import the xyloglucan degradation products.

**Figure 7:**
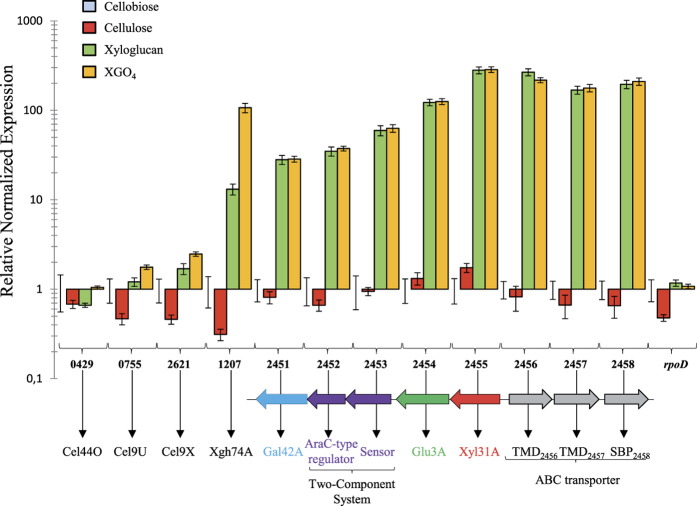
Relative expression of the genes putatively involved in xyloglucan utilization in *R. cellulolyticum* grown on different substrates. Total RNAs were prepared from cultures on cellobiose, cellulose, xyloglucan, and XGO_4_ generated from xyloglucan by digestion with Cel9X, and reverse transcribed. The PCR were then performed with the primer pairs listed in supplemental Table S4. For each gene the relative expression on a given growth substrate versus cellobiose is given after standardization with 16S rRNA encoding gene amplifications. The data show the mean and standard errors from three independent biological replicates. The loci numbers, the predicted functions of the gene products and the schematic representation of the divergent clusters of interest are as in Fig. 6.

To investigate the involvement of the various cellulosomal xyloglucanases and the proteins encoded by the divergent clusters in xyloglucan utilization, RT-qPCR was performed on mRNA extracted from cultures of *R. cellulolyticum* grown in cellobiose-, cellulose-, xyloglucan- and XGO_4_-based minimal media. As seen in Fig. 7, the expression levels of the genes encoding Cel9X, Cel9U and Cel44O did not significantly change, whereas the relative expression levels of the Xgh74A-encoding gene was increased 10- and 100-fold higher on xyloglucan and XGO_4_, respectively, compared to cellobiose-grown cultures. This observation indicates that the expression of the Xgh74A-encoding gene is further induced in presence of XGO_4_ compared to xyloglucan, and suggests Xgh74A is likely to play a prominent role during the cellulosomal degradation of the branched polysaccharide. Thirty- to 300- fold higher relative expression levels of all genes located in the two divergent clusters were also observed on xyloglucan- and XGO_4_-grown cultures compared cellobiose-grown cultures, thereby supporting a direct implication of the corresponding gene products in xyloglucan metabolism and/or transport. No significant difference in the expression level was observed for these genes between xyloglucan- and XGO_4_-based medium.

### Import of the xyloglucan oligosaccharides

**Figure 8:**
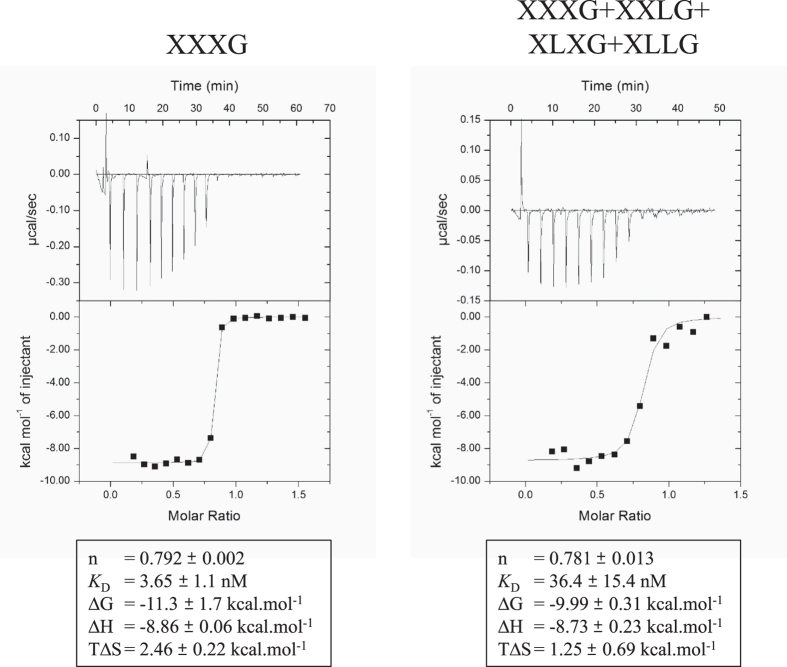
Representative ITC data of the Solute Binding Protein (SBP_2458_) interaction with pure XXXG or with a mixture of XXXG + XLXG + XXLG + XLLG, and affinity. The upper part of each panel shows the raw binding heats and the lower part shows the integrated binding heats minus the dilution control heats fitted to a single-site binding model. The binding parameters are shown at the bottom of each graph, and n designates the number of binding sites on the protein. In the XXXG titration (left panel), the SBP_2458_ was at 6 μM and the ligand at 50 μM. In the XXXG + XLXG + XXLG + XLLG titration (right panel), the SBP_2458_ was at 6 μM and the ligand at a total concentration of 200 μM.

To further examine the role of the ABC-transporter and identify the type(s) of imported oligosaccharides, the SBP_2458_ of the transporter (without the type II leader peptide) was overproduced in the cytoplasm of *E. coli*, and purified. Its capacity to bind various saccharides was explored by IsoThermal microCalorimetry (ITC). SBP_2458_ failed to interact with glucose, xylose, galactose, cellotetraose, cellotriose and isoprimeverose (supplemental Fig. S5). In contrast, as shown in Fig. 8, SBP_2458_ exhibited a very high affinity for XXXG, with an estimated *K*_D_ value of 3.65 ± 1.10 nM. A 10-fold higher *K*_D_ value was determined for a commercial mixture of XXXG, XLXG, XXLG and XLLG, suggesting the ABC-transporter can also efficiently import xyloglucan oligosaccharides carrying galactosyl decorations, but favors XXXG.

The ABC-transporter appeared to be essential for xyloglucan utilization since interruption of the gene encoding SBP_2458_ using a type II intron^41^ (Fig. 6) led to a recombinant strain, named MTL2458, unable to grow on xyloglucan-based medium (supplemental Fig. S6b), whereas the growth on cellobiose-based medium is unaltered (supplemental Fig. S6a). Thus, the phenotype of the mutant strain provides indirect evidence that the identified ABC-transporter is the only one in charge of the import of XGO_4_ in *R. cellulolyticum*, and that XGO_4_ have to be imported in the cytoplasm for their depolymerization into assimilable sugars.

### Cytoplasmic degradation of the xyloglucan oligosaccharides

To explore the ensuing cytoplasmic degradation of XGO_4_, the putative α-xylosidase Xyl31A, β-glucosidase Glu3A and β-galactosidase Gal42A (Fig. 3) were overproduced in *E. coli* and purified.

As expected, Xyl31A was found to efficiently hydrolyze pNP-α-D-xyloside (pNPαXyl; Fig. 1, Table 2) and isoprimeverose, whereas Gal42A exhibited an elevated activity on pNP-β-D-galactoside (pNPβGal). The activity pattern of the β-glucosidase Glu3A was, however, less straightforward. Although elevated *k*_cat_ values were determined on pNP-β-D-glucoside (pNPβGlu) and cellobiose, the enzymatic activity is also characterized by high *K*_m_ values, thereby leading to low catalytic efficiencies on these substrates (Table 2). The β-glucosidase, in contrast to typical β-glucosidases, therefore displays a weak activity on cellobiose as well as on longer cellooligosaccharides (supplemental Table S2).

**Figure 9:**
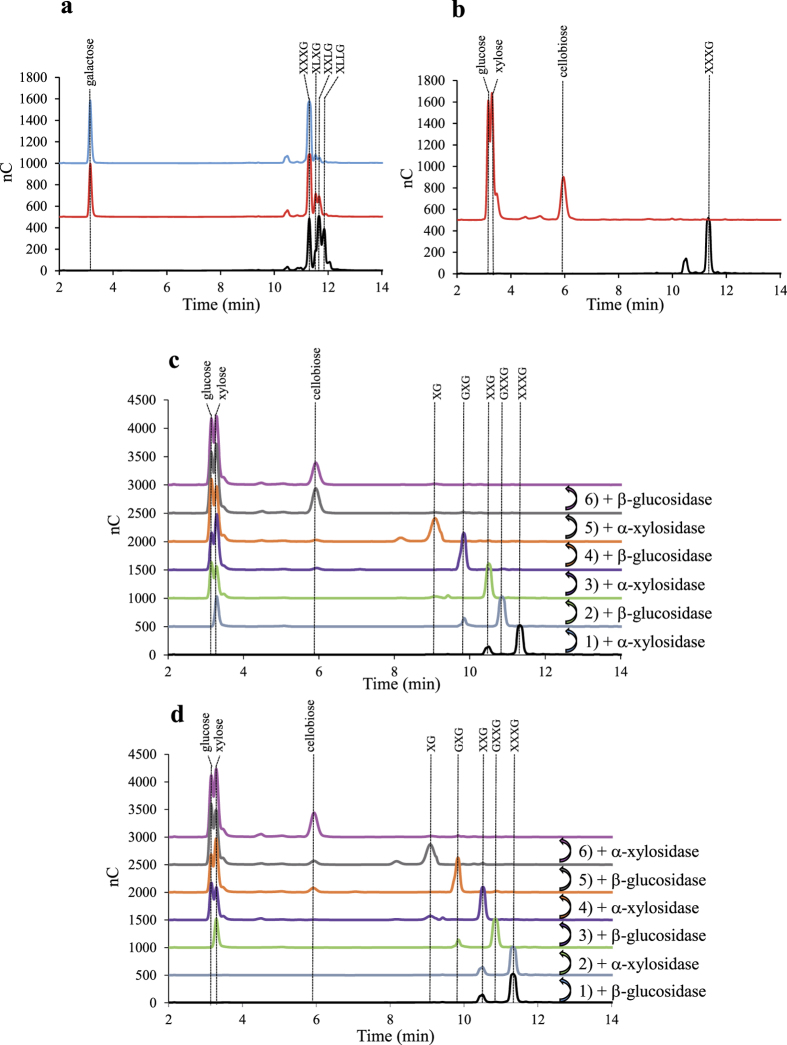
Concerted action of β-galactosidase, α-xylosidase and β-glucosidase on xyloglucan oligosaccharides, analyzed by HPAEC-PAD. (**a**) Activity of the β-galactosidase Gal42A: a mixture of XXXG, XLXG, XXLG and XLLG (see Fig. 1 for xyloglucan abbreviations) at a total concentration of 1 mM was incubated at 37 °C with 0 (black), or 1 μM of Gal42A for 30 min (red) or 270 min (blue). (**b**) Simultaneous degradation of 1 mM XXXG by a mixture of 1 μM of α-xylosidase Xyl31A + 1 μM of β-glucosidase Glu3A. The substrate was incubated for 30 min at 37 °C with no enzyme (black), or with the mixture of Xyl31A and β-glucosidase. (**c**) Sequential degradation of XXXG by Xyl31A and Glu3A, starting with a 30-min incubation with 1 μM of Xyl31A. The sample was afterwards heated at 100 °C for 5 minutes to stop the enzymatic reaction, and an aliquot was analyzed by HPAEC-PAD, prior addition of 1 μM Glu3A, and so on. (**d**) Sequential degradation of XXXG by Xyl31A and Glu3A, starting with a 30-min incubation with the 1 μM Glu3A. The obtained mono and oligosaccharides in each case are shown on top of each corresponding peak. The identification of XG, GXG, XXG, GXXG (which are not commercially available) was performed by mass spectrometry (see supplemental Table S3).

The cytoplasmic enzymes were also assayed on XGO_4_. As shown in Fig. 9a, 1 μM of the GH42 family β-galactosidase rapidly hydrolyzed the β-galactosyl side chains of a commercial mixture of XGO_4_ at 1 mM, which was almost completely converted into galactose and XXXG within 30 min. This result indicates that the enzyme does not require any preliminary action of the α-xylosidase and/or the β-glucosidase to fully remove the galactosyl side chains.

Similarly, the oligosaccharide XXXG at 1 mM was completely depolymerized into 3 mM xylose, 2 mM glucose and 1 mM cellobiose within 30 min by an equimolar mixture of 1 μM α-xylosidase and β-glucosidase (Fig. 9b). Prolonged incubation time up to several hours did not change the proportions of released xylose, glucose and cellobiose.

Sequential degradations of the xyloglucan oligosaccharide were also done by performing successive cycles of 30 minutes of incubation of the oligosaccharide XXXG with either 1 μM of Xyl31A or 1 μM of Glu3A as the first enzyme, followed by a 5-min boiling. A second cycle was then initiated by adding the second enzyme at 1 μM and so on. HPAEC-PAD analyses at the end of each round clearly indicated that the α-xylosidase initiates the depolymerization of XXXG, which is converted into xylose and GXXG (Fig. 9c, supplemental Table S3), the latter oligosaccharide is in turn subsequently hydrolyzed by Glu3A in glucose and XXG. In contrast, for the second sequential degradation starting with the β-glucosidase, no hydrolysis of XXXG was observed during the first round, thereby showing that the Glu3A requires the preliminary action of the α-xylosidase Xyl31A. As expected, at the end of both sequences, the same final products (glucose, xylose and cellobiose) were obtained in the same proportions as that observed for the equimolar mixture of α-xylosidase and β-glucosidase.

**Figure 10:**
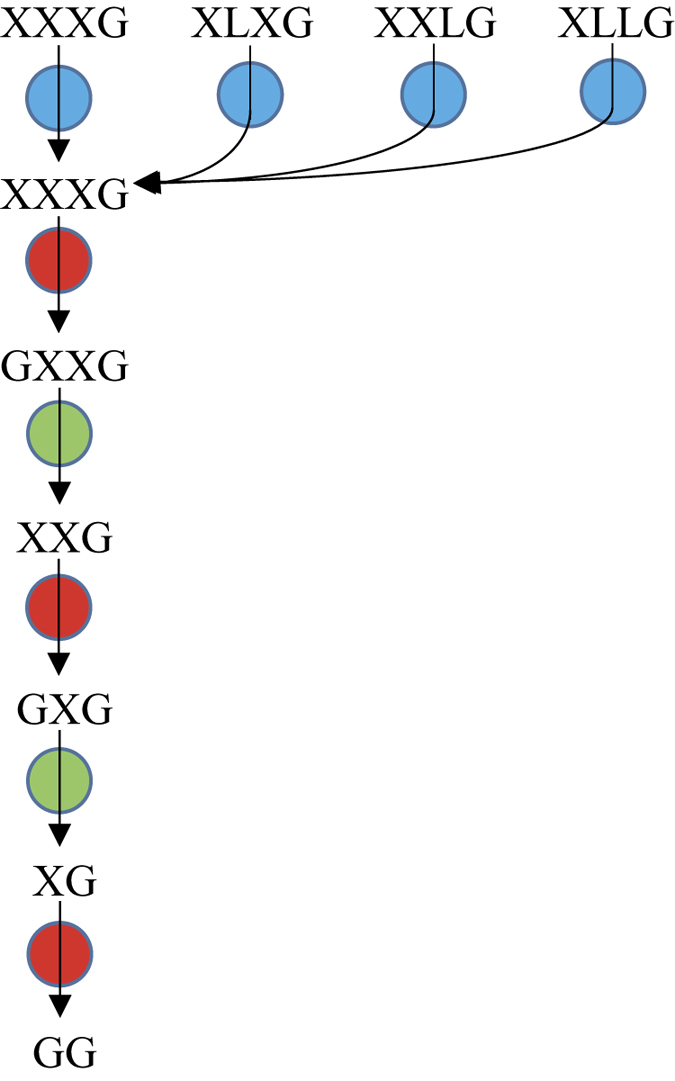
Sequential degradation for the hydrolysis of xyloglucan oligosaccharides by the three cytoplasmic enzymes. β-galactosidase Gal42A, α-xylosidase Xly31A and β-glucosidase Glu3A are represented by blue, red and green circles, respectively. These symbols were selected to facilitate the comparison with the periplasmic degradation pattern proposed earlier for the Gram-negative bacterium *Bacteroides ovatus*^25^. See Fig. 1 for xyloglucan abbreviations.

Altogether these data show that the cytoplasmic depolymerization of XGO_4_ (Fig. 10) taking place in *R. cellulolyticum* diverges to some extent from the model formerly established for the periplasmic degradation performed by the Gram-negative human gut symbiont^25^. Thus, in the cellulosome-producing bacterium, the β-galactosidase Gal42A primarily removes all β-galactosyl units, and the remaining XXXG oligosaccharide is then hydrolyzed from the non-reducing end by the α-xylosidase Xyl31A which removes the α-xylosyl unit, followed by the action of the β-glucosidase Glu3A which is specific of xyloglucan oligosaccharides, and so forth. Another difference concerns the final products which are not only galactose, xylose and glucose but also cellobiose, due to the low catalytic efficiency of Glu3A on the disaccharide.

## Discussion

The degradation and the metabolism of the highly branched polysaccharide xyloglucan has been documented to date for only two Gram-negative bacteria^25^,^26^, though this hemicellulosic polymer is widespread in plant cell walls, especially primary cell walls. Nevertheless, many cellulolytic microorganisms isolated from both aerobic and anaerobic biotopes, were found to produce at least one enzyme classified in GH family 5, 9, 12, 16, 44 or 74 displaying either endo- or exoxyloglucanase activity^31^. Such activities were formerly discovered in the cellulosome-producing bacterium *R. cellulolyticum*^19^, and our data revealed that the identified cellulosomal enzymes displaying xyloglucanase activity not only serve to make the cellulose accessible to the accompanying cellulosomal cellulases, but are involved in the xyloglucan catabolism and associated growth of the bacterium.

**Figure 11:**
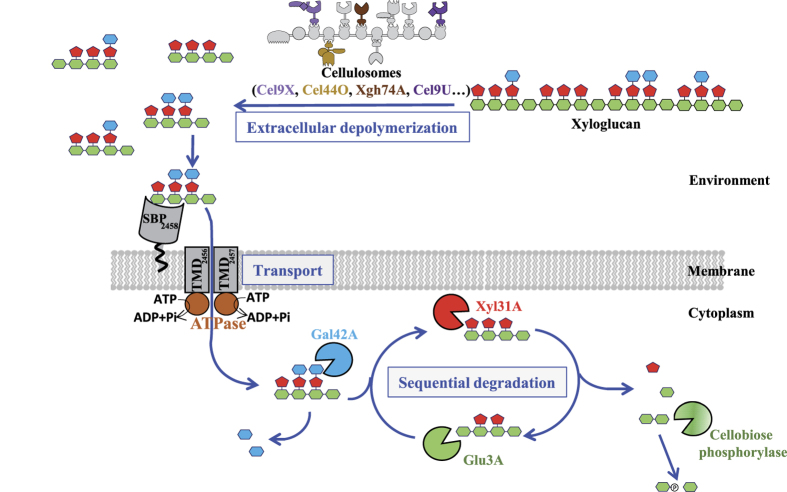
General model for xyloglucan utilization by *R. cellulolyticum*. A cellulosome in charge of the conversion of the polysaccharide into XGO_4_ is shown in light grey and contains the identified cellulosomal enzymes active on xyloglucan Cel9X (light purple), Cel44O (light brown), Xgh74A (dark brown) and Cel9U (dark purple). The oligosaccharides are subsequently imported within the cell through the specific ABC-transporter (dark grey). The next step involves the sequential degradation of the imported XGO_4_ by the β-galactosidase Gal42A (blue partial circle), the α-xylosidase Xyl31A (red partial circle) and the β-glucosidase Glu3A (green partial circle) into galactose (blue diamond), xylose (red diamond), glucose (green diamond) and cellobiose in the cytoplasm. Cellobiose would then be converted into glucose-1-phosphate and glucose by a cellobiose phosphorylase yet to be identified (gradient green partial circle).

On the basis of all of our data, a general model for xyloglucan utilization involving three distinct steps is proposed in Fig. 11. The cellulosomes harboring Cel9X, Cel9U, Cel44O and Xgh74A are in charge of the extracellular degradation of xyloglucan into XGO_4_. However, the list of cellulosomal enzymes displaying high activity on xyloglucan provided in the present report may not be comprehensive, as other cellulosomal glycoside hydrolases yet to be discovered may also exhibit significant activity on this branched polysaccharide. It should also be noted that a non-cellulosomal GH9 cellulase, Cel9W, was formerly shown to display some modest but detectable activity on tamarind xyloglucan^19^. One could thus hypothesize that the xyloglucan oligosaccharides released by Cel9W from the branched polysaccharide may participate to the induction of the expression of the Xgh74A-encoding gene, whose expression level was shown to be ten-fold higher on XGO_4_, compared to xyloglucan (Fig. 7).

The second step involves the recognition and the transport across the membrane of the XGO_4_ by a highly specialized ABC-transporter, the expression of the corresponding genes being specifically induced by xyloglucan and XGO_4_. The transporter is composed of two TransMembrane Domains (TMD_2456_ and TMD_2457_) likely to form a heterodimer transmembrane channel, and a family-1 Solute Binding Protein (SBP_2458_) exhibiting a very high affinity for xyloglucan oligosaccharides, especially for XXXG. To our knowledge, this is the first ABC-transporter ever described as a xyloglucan oligosaccharides importer, and the observed affinity of SBP_2458_ for XXXG is among the highest ever reported for a solute binding protein and its glycosidic ligand^42^,^43^. The ABC-transporter, which was shown to be essential for growth on xyloglucan, would thus import voluminous oligosaccharides composed of up to 9 monosaccharides (in the case of XLLG) at a rather low energy cost.

The final step implies the sequential degradation of the imported XGO_4_ in the cytoplasm, beginning with the β-galactosidase Gal42A which removes all β-galactosyl units thus converting XGO_4_ into XXXG (and galactose), similarly to the GH35 β-galactosidase from *C. japonicus*^26^. XXXG is subsequently subjected to successive rounds of hydrolysis by the α-xylosidase Xyl31A and the β-glucosidase Glu3A. In this model, the end products are not only galactose, xylose and glucose as reported for the periplasmic degradation of XGO_4_ by Gram-negative bacteria^25^, but also cellobiose. The disaccharide is presumably rapidly metabolized by *R. cellulolyticum* whose genome encodes at least one cytoplasmic cellobiose phosphorylase that may convert the cellobiose generated by the depolymerization of the XGO_4_, into glucose-1-phosphate and glucose^22^, thereby saving one ATP molecule when the phosphorylated hexose enters the glycolytic pathway.

The present study was restricted to the metabolism of tamarind xyloglucan but other xyloglucans exhibit another repeated motif, XXGG, or harbor additional or alternative decorations such as α-1,2-linked fucosyl residues to the first galactosyl residue in XLLG (and XXLG) or an α-1,2-linked arabinofuranosyl residues to the xylosyl residues in XXGG^4^. Nevertheless, the genome of *R. cellulolyticum* encodes two putative GH95 α-fucosidases: a cytoplasmic and a secreted cellulosomal catalytic enzyme. Similarly, 10 genes were predicted to encode α-arabinofuranosidases^22^: seven GH43, one GH51 and two GH62. Four of these putative α-arabinofuranosidases are presumed to be cytoplasmic (three GH43 and the GH51), whereas four other enzymes (two GH43 and two GH62) are appended with a dockerin module and expected to participate to the extracellular cellulosomes^22^,^23^,^44^. These observations suggest that *R. cellulolyticum* is probably well equipped for hydrolyzing either extra- or intracellularly the variable decorations found in other types of xyloglucans. Nonetheless, the capacity of the identified cellulosomal enzymes displaying elevated activity on tamarind xyloglucan (Cel9U, Cel9X, Cel44O and Xgh74A) to depolymerize these alternative xyloglucans, and the ability of the ABC-transporter to import XGO_4_ decorated with α-1,2-fucosyl or α-1,2-arabinofuranosyl residues will need to be explored.

Conversely to *R. cellulolyticum*, the genome of the model thermophilic cellulosome-producing bacterium *R. thermocellum* does not encode any GH31 α-xylosidase or GH42 β-galactosidase. Furthermore, the genes coding for an ABC-transporter similar to that described in the present report are also missing, thereby suggesting the thermophilic bacterium cannot metabolize xyloglucan, though its cellulosomes contain some enzymes experimentally demonstrated to be active on xyloglucan^35^,^36^,^45^,^46^. Consistently we observed the inability of *R. thermocellum* to grow on xyloglucan-based (3.5 g/L) rich medium, even after three weeks of incubation at 55 °C, whereas the stationary phase is reached within 24 h on the same medium but containing 3.5 g/L cellobiose as the carbon source. Based on these observations, it is hypothesized that the cellulosomal xyloglucanases in *R. thermocellum* mainly help the accompanying cellulases in the complex to access the cellulose microfibrils, as was previously suggested for cellulosomal xylanases^47^.

**Figure 12:**
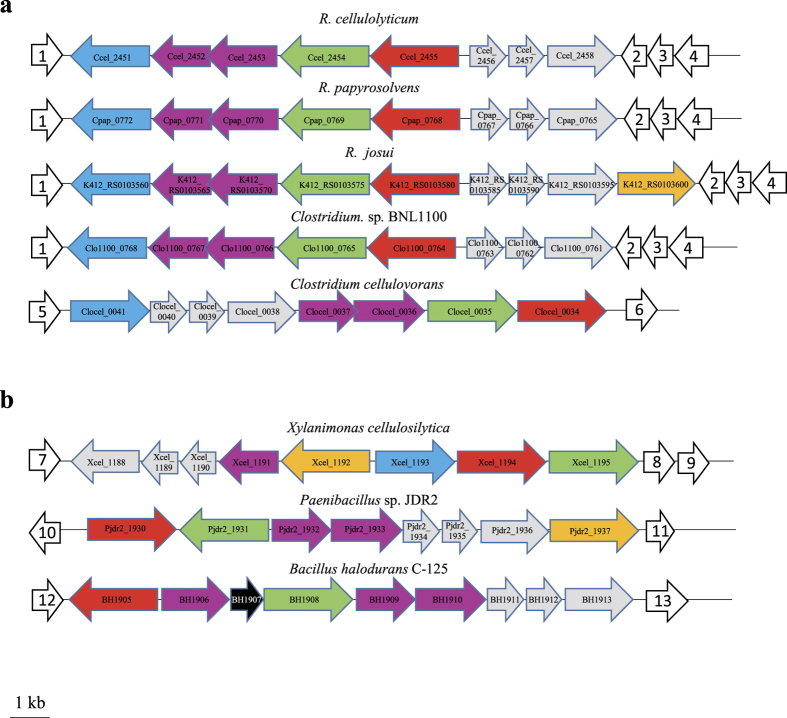
Occurrence of the similar gene clusters involved in the transport and the metabolism of XGO_4_ in (**a**) other cellulosome-producing *firmicutes* and in (**b**) some more distantly related procaryotes. The α-xylosidase, β-glucosidase and β-galactosidase-encoding genes are shown in red, green and blue, respectively. The genes encoding the two-component system are represented in purple, and the genes encoding the ABC-transporter are shown in grey. The α-fucosidase-encoding genes are shown in yellow. The gene in black corresponds to a gene of unknown function. The corresponding locus tags are indicated in the genes. The flanking genes are shown in white, and numbered from 1 to 13. Genes 1, 2, 3 and 4 encode putative tryptophane synthase subunit β, hypothetical protein, hypothetical protein and acyl ACP thioesterase, respectively. Genes 5 and 6 code for putative phosphodiestase and formate/nitrite transporter, respectively. Genes 7, 8 and 9 code for putative hypothetical protein, hypothetical protein and methyltransferase, respectively. Genes numbered 10 and 11 encode putative transcriptional regulator and hypothetical protein. Genes 12 and 13 encode putative stage II sporulation protein P and spermine synthase, respectively.

In contrast, as shown in Fig. 12a, most mesophilic cellulosome-producing bacteria and the moderate thermophile *R. josui*, exhibit highly similar gene clusters devoted to cytoplasmic degradation and transport of XGO_4_. The genome of these bacteria also encodes cellulosomal or extracellular enzymes displaying modular organizations comparable to that of Cel9U, Cel9X, Cel44O and Xgh74A. Thus, the mechanisms involved in xyloglucan utilization described in this report may be widespread among mesophilic organisms synthesizing cellulosomes but also, as shown in the examples reported in Fig. 12b, in more distantly related Gram-positive bacteria that display comparable gene clusters.

All the clusters reported in Fig. 12a also include two genes encoding a two-component system, encompassing a sensor and AraC-like transcriptional regulator, whose expression is also strongly induced by xyloglucan and XGO_4_ in the case of *R. cellulolyticum* (Fig. 7). Its probable implication in the regulation of the surrounding genes encoding the ABC-transporter (as formerly shown for a two-component system in *Geobacillus stearothermophilus*^48^) and the cytoplasmic enzymes will need to be examined in the future.

In a recent study, a polysaccharide utilization locus named XyGUL was found to constitute a genetic marker of xyloglucan catabolism in Gram-negative human gut symbiont *Bacteroidetes*^25^. The divergent gene clusters described in the present report and forming a xyloglucan oligosaccharide utilization locus may provide a promising additional genetic marker of xyloglucan catabolism by Gram-positive bacteria.

## Methods

### Bacterial strains, plasmids and media

Genomic DNA from *Ruminiclostridium cellulolyticum* H10 ATCC 35319 was used as a template for amplification by PCR of the DNA encoding the mature forms of the various proteins. A list of the primers used in this study is provided in supplemental Table S4. The amplicons were cloned in pET28a(+) at NcoI/XhoI sites, except for the gene encoding the SBP which was cloned in pET22b(+) at NdeI/XhoI sites. In all cases, six His codons were introduced at the 3′ extremity of the coding sequences. Positive clones were verified by DNA sequencing. pET28a-Cel9Xt and pET28a-9X-44O which encode Cel9X appended with the dockerin module of CelS^49^ from *R. thermocellum* and the fusion of the two enzymes Cel9X and Cel44O (Fig. 3) were obtained by successive overlap extension PCRs. The DNA encoding the C-terminal region of Cel9X core enzyme was amplified from pET28a-Cel9X^19^ using primers X830f/XdokeTR and the DNA coding for the dockerin of CelS was amplified from pET-9Gt^13^ using the primers XdokeTF/pETrev. The resulting overlapping fragments were mixed and a combined fragment was synthesized using the external primers. The fragment was subsequently cloned at EcoRI/XhoI sites in pET28-Cel9X thereby generating pET28a-Cel9Xt. The DNAs encoding Cel9X and Cel44O were amplified from pET28a-Cel9Xc and pET28a-Cel44O using primers 9Xf/9X44Or and 9X44Of/44Or for *cel9X* and *cel44O* amplification, respectively. The resulting overlapping fragments were mixed. The combined fragment was synthesized using the external primers and was subsequently cloned at NcoI/XhoI sites in pET28a, thus generating pET28a-9X-44O.

The *Escherichia coli* BL21(DE3) strain was used as the production strain and was grown at 37 °C in lysogenic broth supplemented with appropriate antibiotics. *R. cellulolyticum* strain was grown anaerobically at 32 °C on basal medium^27^ supplemented with either 2 g/L cellobiose, 5 g/L crystalline cellulose Sigmacell20, 3.5 g/L tamarind xyloglucan or 3.5 g/L xyloglucan oligosaccharides XGO_4_, obtained after digestion of 3.5 g/L tamarind xyloglucan by 0.5 μM of Cel9X for 24 h at 37 °C in 18 g/L MOPS, 2.17 g/L KH_2_PO_4_, pH 6.0. The total conversion into XGO_4_ was verified by HPAEC-PAD, as formerly described^19^. *R. thermocellum* (ATCC 27405) strain was grown anaerobically at 55 °C on basal rich medium^50^ supplemented with either 3.5 g/L cellobiose or 3.5 g/L xyloglucan.

### *R. cellulolyticum* mutant strain construction

The disruption of the target gene at locus Ccel_2458 encoding SBP_2458_ was performed using the ClosTron technology^41^. The primers IBS-SBP, EBS1d-SBP and EBS2-SBP used for retargeting L1.LtrB intron carried by pMTL007^41^ (supplemental Table S4) were designed with the Perutka algorithm available at Clostron website (http://www.clostron.com/). A 353-bp amplicon was obtained by overlapping PCR using IBS-SBP, EBS1d-SBP, EBS2-SBP and universal EBS, and cloned at HindIII/BsrGI sites in pMTL007, thereby generating pMTL007-SBP. *R. cellulolyticum* was electrotransformed as previously described with pMTL007-SBP treated with MspI methylase^51^,^52^, and thiamphenicol resistant clones carrying replicative pMTL007-SBP were selected. In a second step, the integration event was selected in erythromycin-containing basal medium after induction with 1 mM IsoPropyl Thio-β-D-Galactoside (IPTG). The antisens integration of the intron at Ccel_2458 (position 707/708) was verified by PCR using the primer pairs SBPf/ramR, SBPr/ramF and SBPf/SBPr. The modified *R. cellulolyticum* strain was named MTL2458.

### Protein production and purification

Purifications of Cel9X, Cel9U and Scaf4 were formerly described^19^. The BL21(DE3) overproducing strains were grown in toxin flasks at 37 °C in lysogenic broth supplemented with glycerol (12 g/L) and the appropriate antibiotic until *A*_600_ = 1.5. The cultures were cooled down and induction of the expression was performed overnight at 16 °C with 50 μM IPTG for the strains carrying pET28a-Gal42A, pET28a-Glu3A and pET28a-Xyl31A, 100 μM IPTG for the strains carrying pET28a-Cel44O, pET28a-Cel9Xt, pET28a-Xgh74A and pET22b-SBP, and 200 μM IPTG for the strain carrying pET28a-9X-44O. The proteins of interest were purified using a two-step procedure essentially as formerly described^19^. In the case of Gal42A, a third purification step was necessary. After the anion exchange chromatography, the fractions containing Gal42A were concentrated by ultrafiltration to 2 mL and purification was achieved by gel filtration on a Superdex 200 10/300 GL resin equilibrated in 50 mM potassium phosphate pH 7.0, 150 mM NaCl.

### Enzymatic assays

Activity assays on CMC medium viscosity, PASC, microcrystalline cellulose Avicel, tamarind xyloglucan, cellooligosaccharides, *p*NPβGlu, *p*NPβGal, *p*NPαXyl were performed as previously described^19^ with final enzyme concentrations ranging from 1 nM to 1 μM. Cellulosomes, purified from a cellulose-grown culture as previously described^8^, were assayed on 3.5 g/L tamarind xyloglucan in 20 mM Tris maleate, pH 6.0, 1 mM CaCl_2_, 0.01% NaN_3_ (w/v) by mixing the substrate solution with 6 mg/L of cellulosomes at 37 °C for 3 h. Twenty-μL aliquots were mixed with 180 μL of distilled water and 50 μL of 0.5 M NaOH prior analysis by HPAEC-PAD. Activity on isoprimeverose was performed in 20 mM sodium phosphate, pH 6.0, 0.01% NaN_3_ (w/v) by mixing 80 μL of a substrate solution at 0.9 mM with 0.8 μL of an appropriate dilution of enzyme at 37 °C. At specific intervals, 20-μL aliquots were pipetted and prepared as described above for HPAEC-PAD. The sequential degradation of pure XXXG and XGO_4_ commercial mixture by cytoplasmic enzymes was monitored by incubating 0.2 mL of substrate solution at 1 mM with 1 μM of enzyme in 20 mM sodium phosphate, pH 6.0, 0.01% NaN_3_ (w/v) at 37 °C for 30 min. At the end of the incubation, aliquots were pipetted for HPAEC-PAD and MALDI-TOF analyses, and the remainder sample was boiled for 5 min prior adding the next enzyme. Activity of the β-glucosidase on the end products generated by Cel9X or Cel44O on xyloglucan was performed as follows: One mL of 3.5 g/L tamarind xyloglucan in 20 mM sodium phosphate, pH 6.0, 0.01% NaN_3_ (w/v) was mixed with 0.5 μM of Cel9X or Cel44O at 37 °C. After 24 h of incubation, end products released by the enzymes were verified by HPAEC-PAD. The sample was then boiled for 5 min, and incubated with 1 μM β-glucosidase at 37 °C. Twenty-μL aliquots were pipetted after 30 min and 2 h of incubation and prepared for HPAEC-PAD analyses. Viscosimetric assays on xyloglucan were performed as previously described^19^.

### RNA Isolation and Reverse Transcription in cDNA

Total RNA was isolated using High Pure RNA Isolation kit (Roche Applied Science) from *R. cellulolyticum* wild type strain grown until mid- to late- exponential phase in minimal medium containing either cellobiose (2 g/L), cellulose (5 g/L), xyloglucan (3.5 g/L) or XGO_4_ (3.5 g/L) as the sole carbon source. DNAse I treatment (Ambion, Life Technologies) was used to remove the contaminating DNA. cDNA synthesis was performed using SuperScript III reverse transcriptase (Invitrogen) and random primers (Invitrogen) from 200 ng of total RNA.

### Quantitative Polymerase Chain Reaction (qPCR)

Quantification of cDNA was carried out with the SsoFast^TM^ EvaGreen^®^ Supermix (Bio-Rad) according to the manufacturer’s protocol. Complementary DNA was mixed with 0.5 μM of each pair of specific primers in 15 μL final volume. These specific primers (supplemental Table S4) were designed using the tool Primer-Blast (V 3). Real-time PCR was carried out on a CFX96 Real-Time PCR detection system (Bio-Rad) and the software CFX Manager 3.1. Thermal cycler was programmed for an initial step at 98 °C for 2 min, followed by 45 cycles comprising denaturation step at 98 °C for 5 s, hybridization step at 56 °C for 10 s and elongation step at 72 °C for 1 s. Specificity of accumulated products was verified by using the melting-curve analysis from 65 °C to 95 °C, with fluorescence measurement every 0.5 °C. Results with only a single peak at the right temperature were considered. The Relative Expression Software Tool (REST) was used to calculate the relative expression of each gene in each condition using 16S-RNA encoding gene. Quantification was performed in duplicate on each cDNA preparation.

### Matrix-Assisted Laser Desorption/Ionization-Time-Of-Flight (MALDI-TOF) analysis of xyloglucan oligosaccharides

Xyloglucan oligosaccharides released after 24 h of incubation at 37 °C by 0.2 μM of Cel44O, 0.1 μM of Cel9X and 0.1 μM of the fusion 9X-44O on 3.5 g/L tamarind xyloglucan, were subjected to MALDI-TOF analyses. Similarly, samples obtained during the sequential degradation of XXXG by 30-minute incubations at 37 °C with either 1 μM of α-xylosidase or 1 μM of β-glucosidase were analyzed using the same procedure. MALDI-TOF analyses were performed on a Microflex II mass spectrometer (Bruker Daltonics) as previously described^53^. One μL of matrix (10 mg of 2,5-Dihydroxybenzoic acid in 1 mL of CH_3_CN/H_2_O/50/50 (v/v), 0.1% formic acid (v/v)) was added to 1 μL of sample (100 pmoles) in the same solution. In reflectron positive mode used for acquisition (mass range 160–3000 Da), accuracy was less than 50 ppm.

### Isothermal Titration Calorimetry (ITC)

Thermodynamic parameters were estimated by isothermal titration calorimetry (ITC) using a MicroCal iTC200 (Malvern-Microcal) microcalorimeter. All experiments were carried out at 20 °C in a buffer containing 150 mM NaCl and 25 mM HEPES pH 7.0.

For the interaction with XXXG several measurements were done with the Solute Binding Protein (SBP_2458_) added in the 200.4 μL cell at a concentration between 6 and 40 μM and the ligand between 50 and 400 μM. For XGO_4_ commercial mixture, the concentration of SBP ranged between 6 and 12 μM and the ligand between 50 and 200 μM.

For glucose, galactose, xylose, cellotriose, cellotetraose and isoprimeverose for which no interaction was detected, the concentration of the protein in the cell was 50 μM and the concentration of the different sugars 500 μM. For isoprimeverose, an experiment was also done with protein concentration in the cell at 40 μM and the ligand at 4 mM (supplemental Fig. S5).

A theoretical titration curve using Origin, the software supplied by Microcal, was fit to the experimental data. This software uses the relationship between the heat generated by each injection and ΔH° (enthalpy change in kcal.mol^−1^), *K*_A_ (association binding constant in M^−1^), n (number of binding sites per monomer), total protein concentration, and free and total ligand concentrations. The variation in the entropy (ΔS° in cal.mol^−1^.deg^−1^) of each binding reaction was inferred from the variation in the free energy (ΔG°), where this latter was calculated from the following relation: ΔG° = −RTLn(*K*_A_). For each ligand, experiments were repeated at least three times.

## Additional Information

**How to cite this article**: Ravachol, J. *et al.* Mechanisms involved in xyloglucan catabolism by the cellulosome-producing bacterium *Ruminiclostridium cellulolyticum*. *Sci. Rep.* **6**, 22770; doi: 10.1038/srep22770 (2016).

## Supplementary Material

Supplementary Information

## Figures and Tables

**Table 1:** Specific activities of wild type and engineered cellulosomal enzymes on cellulosic substrates and xyloglucan.

Substrate (concentration)	Enzyme				
	Cel9X^a^	Cel9U^a^	Cel44O	9X-44O	Xgh74A
CMC (10 g/L)	–	6,666 ± 310	2,613 ± 627^b^	2,584 ± 511	1,057 ± 7
PASC (3.5 g/L)	–	736 ± 6.5	393 ± 96	413 ± 48	55 ± 3.5
Avicel (3.5 g/L)	–	44.9 ± 0.4^c^	38 ± 2	31 ± 0.3	10.7 ± 0.7
Xyloglucan (3.5 g/L)	1,250 ± 85	1,069 ± 243	922 ± 95	1,340 ± 14.4	2,160 ± 126

^a^The data are from ref. 19.

^b^The data show the mean and standard deviation of 2 to 4 replicates.

^c^Values are given in μmol of products released per μmol of enzyme × min^−1^, except for Avicel where the values are in μmol of products released after 24 h of incubation with 0.1 μmol of enzyme.

**Table 2:** Activities of α-xylosidase, β-glucosidase and β-galactosidase on various oligosaccharides.

substrate	Enzyme								
	α-xylosidase			β-glucosidase			β-galactosidase		
	*k*_*cat*_	*K*_m_	*k*_*cat*_/*K*_m_	*k*_*cat*_	*K*_m_	*k*_*cat*_/*K*_m_	*k*_*cat*_	*K*_m_	*k*_*cat*_/*K*_m_
*p*NPα Xyl	12^a^ ± 1.2	1.29^b^ ± 0.15	9.3^c^	Not detected			Not detected		
*p*NPβGlu	Not detected			1,350 ± 164	44.6 ± 19	30	Not detected		
*p*NPβGal	Not detected			0.067 ± 0.001^d^			4,800 ± 320	1.56 ± 0.3	3,077
Isoprimeverose	734 ± 175	1.9 ± 0.5	386	Not detected			Not detected		
Cellobiose				412 ± 44	42.5 ± 5.2	9.7			

^a^Values are given in μmol of products released per μmol of enzyme × min^−1^.

^b^Values are given in mM.

^c^Values are given in μmol of products released per μmol of enzyme × min^−1^ × mM^−1^.

^d^*p*NPβGal was at 3.3 mM and the value of the activity is given in μmol of products released per μmol of enzyme × min^−1^. The data show the mean and standard deviation of two independent experiments.
