# Supplementary material for: Mechanisms involved in xyloglucan catabolism by the cellulosome-producing bacterium Ruminiclostridium cellulolyticum
Source: Sci Rep. 2016 Mar 7;6:22770. doi: 10.1038/srep22770 (PMC4780118; doi:10.1038/srep22770)

Supplementary information

Mechanisms involved in xyloglucan catabolism by the cellulosome-producing bacterium *Ruminiclostridium cellulolyticum*

Julie Ravachol[1], Pascale de Philip[1], Romain Borne[1], Pascal Mansuelle[2], María J. Maté[3], Stéphanie Perret[1] and Henri-Pierre Fierobe[1]*

[1]Aix-Marseille Université-CNRS, Laboratoire de Chimie Bactérienne, UMR7283, IMM, 31 chemin Joseph Aiguier, F-13402 Marseille, France.

[2]Plate-forme de Protéomique, IMM, 31 chemin Joseph Aiguier, F-13402 Marseille, France.

[3]Aix-Marseille Université-CNRS, Laboratoire d'Architecture et Fonction des Macromolécules Biologiques, UMR7257, Parc Technologique de Luminy, F-13288 Marseille, France.

[*]To whom correspondence should be sent: hpfierob@imm.cnrs.fr

Supplementary Table S1: Kinetic parameters of the hydrolytic activities of Cel9U, Cel9X, Cel44O and Xgh74A on tamarind xyloglucan

Enzyme	k_{cat}[a]	K_m[b]	k_{cat}/K_m[c]
Cel9U[d]	2,302 ± 157	7.5 ± 0.15	307
Cel9X[d]	6,560 ± 231	8.4 ± 0.4	781
Cel44O	970 ± 38	0.21 ± 0.06	4,619
Xgh74A	2,304 ± 78	0.37 ± 0.01	6,227

[a]values are given in µmol of products released per µmol of enzyme x min^{-1}.
[b]values are given in g x L^{-1} of xyloglucan.
[c]values are given in µmol of products released per µmol of enzyme x min^{-1}L x g^{-1}.
[d]data are from reference[19].

Supplementary Table S2: Specific activity of the β-glucosidase GH3A on 1 mM cellooligosaccharides.

Substrate	Specific activity[a]
Cellobiose	9.0 ± 0.20[b]
Cellotriose	12.4 ± 1.3
Cellotetraose	11.6 ± 1.3
Cellopentaose	14.0 ± 2.3
Cellohexaose	10.7 ± 0.6

[a]values are given in µmol of products released per µmol of enzyme x min^{-1}.
[b]the data show the mean and standard deviation of two replicates.

Supplementary Table S3: MALDI-TOF analyses of the products released during the sequential degradation of XXXG by successive rounds of incubation with either the α-xylosidase or the β-glucosidase.

Corresponding steps in Figures 9c or 9d	Composition	Expected m/z $[M+Na]^+$	Observed m/z $[M+Na]^+$
Substrate	XXXG[a]	1,085.338	1,085.306
9c step 1 (+ α-xylosidase)	GXXG	953.296	953.322
9c step 2 (+ β-glucosidase)	XXG	791.243	791.237
9c step 3 (+ α-xylosidase)	GXG	659.201	659.137
9c step 4 (+ β-glucosidase)	XG	497.148	497.131
Substrate	XXXG	1,085.338	1,085.306
9d step 1 (+β-glucosidase)	XXXG	1,085.338	1,085.290
9d step 2 (+α-xylosidase)	GXXG	953.296	953.209
9d step 3 (+β-glucosidase)	XXG	791.243	791.198
9d step 4 (+α-xylosidase)	GXG	659.201	659.139
9d step 5 (+β-glucosidase)	XG	497.148	497.081

[a]see Fig. 1 for xyloglucan motif abbreviations.

Supplementary Table S4: list of primers used

Primers used for overexpression in *E. coli* of selected genes

Protein (gene locus)	Primer name	5'-3' primer sequence
**Cel44O (Ccel_0429)**	44Of	AAAAAACCATGGCTGCCAGTGACGCAATAAACG
	44Or	AAAAAACTCGAGCCAATCAATTGAATCAACATACATT
**Xgh74A (Ccel_1207)**	Ccel_1207f	TTTTTTCCATGGCAAATGGCCCCGTAAGCGCCCCGTATAATTGGAA
	Ccel_1207r	GGGGGGCTCGAGAAATGAGGTAATTATTCCCAATAAATAAC
**Gal42A (Ccel_2451)**	2451ncof	TTTTTTCCATGGATAAATATATGCCTATTGGTATG
	2451mnf	CTAAAGGATTTCCACGGCGGACGTC
	2451mnr	GACGTCCGCCG TGGAAATCCTTTAG
	2451xhor	TTTTTCTCGAGTATTTTAGCTTTTAGCACTTTTACGTCA
**Glu3A (Ccel_2454)**	2454Ncof	AAAAACCATGGAATACGATCAGATAGATAAAAAAATTGATGAAC
	2454Xhor	AAAAACTCGAGCAGAGCAAGAGCTATAGCTATCG
**Xyl31A (Ccel_2455)**	2455ncof	TATATACCATGGCTAAATTTCTAAATGGATACTGGATGAGT
	2455xhor	TTTTTCTCGAGTAGAATTCTTACTGTATAACTGCCAGTAGA
**SBP_{2458} (Ccel_2458)**	SBPf	AATTCATATGCACCACCACCACCACCACGCTTGTACCAAACCGGGAACCGACAGC
	SBPrev	AATTCTCGAGTTATTTACTGTTTTCTGCAGCCTGCTTGCGGG
**Cel9Xt**	XdokeTf	GCATTCGCCAACAGTAATTTGTCAGGTACTCCTTCTACTAAATTATACGGC
	XdokeTR	GCCGTATAATTTAGTAGAAGGAGTACCTGACAAATTACTGTTGGCGAATGC
	X830f	CCCCCAGGTTCAAGATAGGA
**9X-44O**	9X44Of	CAGTAATTTGTCAGGTGAAAACTCCATAGCTGCCAGTGACGCAATAAACG
	9X44Or	CGTTTATTGCGTCACTGGCAGCTATGGAGTTTTCACCTGACAAATTACTG
	pETrev	ATGTTTGACAGCTTATCATCGA

Primers used for qPCR experiments designed with the Primer-BLAST tool

Protein (gene locus)	Primer name	5'-3' primer sequence
**Gal42A (Ccel_2451)**	2451f	CTCGTGGATGTCGCAAAAGT
	2451r	CACCACTCATGGTTAAGCCG
**Regulator (Ccel_2452)**	2452f	CGGCAAGCTCGGTAAATCTT
	2452r	CGTCCCACAGAAGCAAACAA
**Sensor (Ccel_2453)**	2453f	CAGCACCATGTGGGATAACG
	2453r	CGTATACGGCGTACCAAAGC
**Glu3A (Ccel_2454)**	2454f	TGCTGCCAATAACCAGGAGA
	2454r	TCCGTAATCAACTGACGGCT
**Xyl31A (Ccel_2455)**	2455f	TACGGTTTGGGAGAGCGATT
	2455r	TCCAACGATTCGCCTTCAAC
**TMD_{2456} (Ccel_2456)**	2456f	GAAGTCAAGCGTGGTTGGTT
	2456r	CCACAAACTCCAAAGCGTCA
**TMD_{2457} (Ccel_2457)**	2457f	TGCTTAATCTGGGGCTTCGT
	2457r	CAAGAATGGGAACGCAGAGG
**SBP_{2458} (Ccel_2458)**	2458f	TGGCAAAGCACGGAGATAAC
	2458r	CCTCGGAAGGAGTAATGGGA
**Cel44O (Ccel_0429)**	0429f	AGCACCGTCAGAAAGATGGA
	0429r	AGAGAGCCGGTTCATTGTCA
**Cel9U (Ccel_0755)**	0755f	CGGTAAAGACGGAGGGAAGT
	0755r	ATTGTAATCCCCGGCATCGT
**Xgh74A (Ccel_1207)**	1207f	GAAGCGGTAACGGACTTTGG
	1207r	GGGTTCCATGTTTCTCCTGC
**Cel9X (Ccel_2621)**	2621f	TACTGAGTGGCAGCACGTAA
	2621r	AGCCGGATATGCTCTTTCCT
**RpoD (Ccel_0541)**	RpoDdir	AAACATAGTCAAGAAAGTAGAAAAG
	RpoDrev	CTATACTAACAACCAGCCTTAAG
**16S rRNA (Ccel_R0018)**	16s235F	CTATGTTTCTTGAGTGCCGG
	16s235R	ATACTTATTGTGTTAACTCCGG

Primers used for construction of *R. cellulolyticum* mutant strain MTL2458

Protein (gene locus)	Primer name	5'-3' primer sequence
	IBS-SBP	AAAAAAGCTTATAATTATCCTTACATGCCGCCGTCGTGCGCCCAGATAGGGTG
	EBS1d-SBP	CAGATTGTACAAATGTGGTGATAACAGATAAGTCGCCGTCCCTAACTTACCTTTCTTTGT
	EBS2-SBP	TGAACGCAAGTTTCTAATTTCGGTTGCATGTCGATAGAGGAAAGTGTCT
	EBS	CGAAATTAGAAACTTGCGTTCAGTAAAC
**SBP_{2458} (Ccel_2458)**	SBPf	GGGTGGATTTATTCACTGTTTGA
	SBPr	GGTTGGCAAGAGCGACGGTATGGTG
**Em^R**	ramF	ACGCGTTATATTGATAAAAATAATAATAGTGGG
	ramR	ACGCGTGCGACTCATAGAATTATTTCCTCCCG

Supplementary Figure S1: Growth on minimal medium supplemented with a) tamarind xyloglucan or b) XGO_4 at 3.5 g/L.

The data show the mean of three independent experiments and the bars represent the standard deviation. Cultures on minimal medium supplemented with tamarind xyloglucan (**a**) or XGO_4 (**b**) were used as inoculum (1/20).

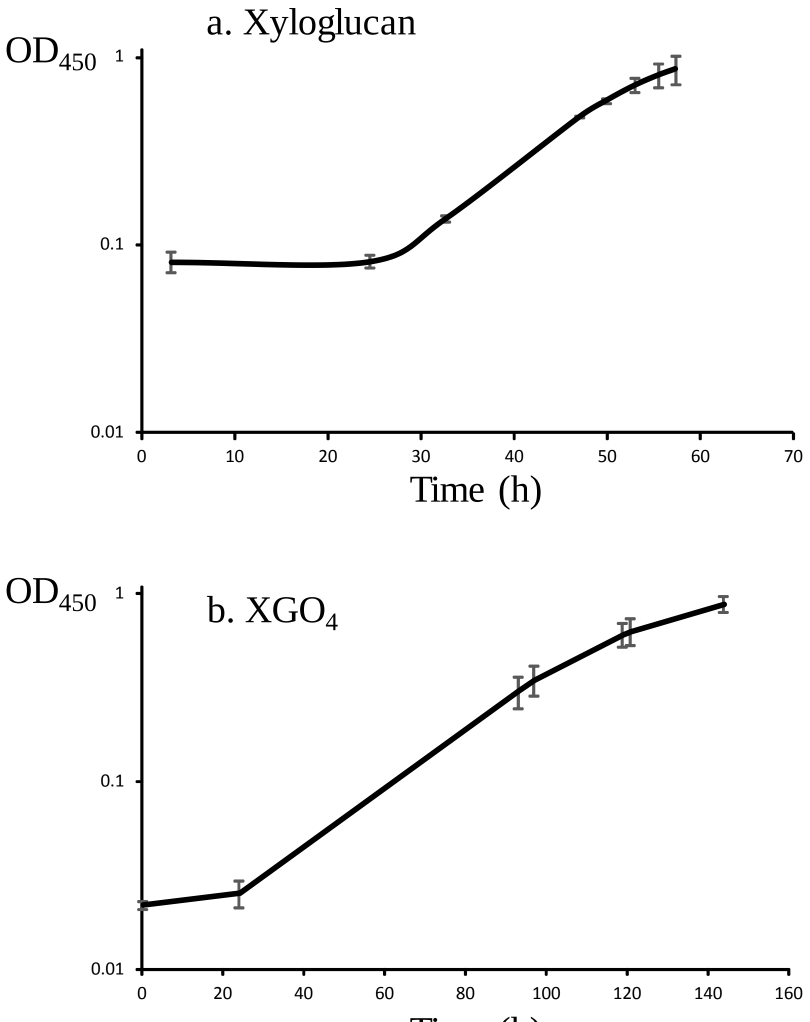

Supplementary Figure S2: Complete degradation of 3.5 g/L xyloglucan by Cel9X.

Xyloglucan was incubated for 24 h at 37°C with no (blue) or 0.5 µM of Cel9X (red). The obtained sugars are indicated on top of each peak. The mixture of XGO_4 oligosaccharides served to prepare the XGO_4-based minimal medium used for growth of *R. cellulolyticum* (Figure S1) and mRNA extraction subsequently used for reverse transcription and quantitative PCR experiments (Figure 7).

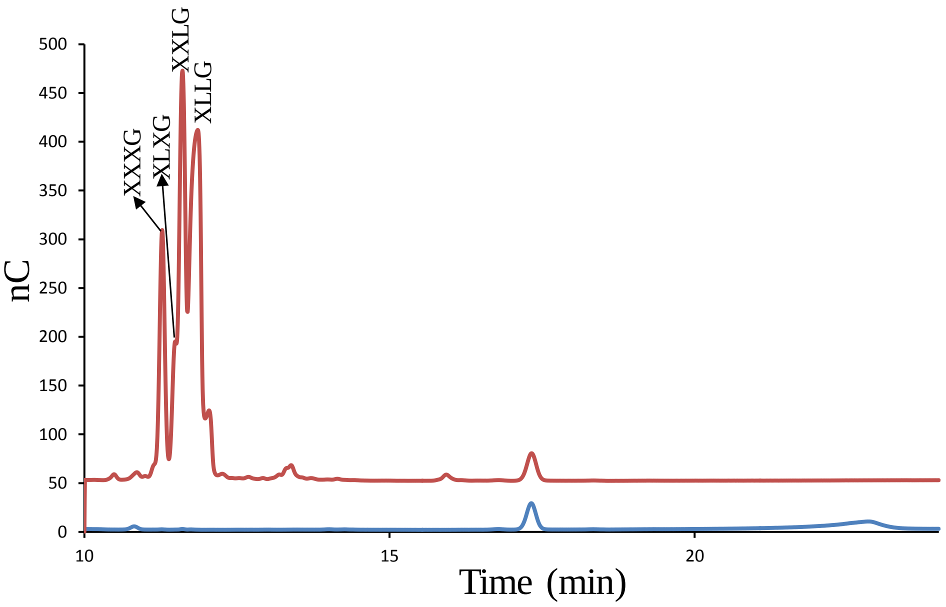

Supplementary Figure S3: Changes in relative xyloglucan viscosity versus reducing sugars released by Cel9X, Cel44O and Xgh74A.

Green line, Cel9X; blue line, Cel44O; red line, Xgh74A. The data show the mean and standard deviation of three independent experiments. The specific viscosity was determined as (($T-T_0)/T_0$) where T and T_0 correspond to the flow time of xyloglucan with enzyme and the flow time of buffer, respectively.

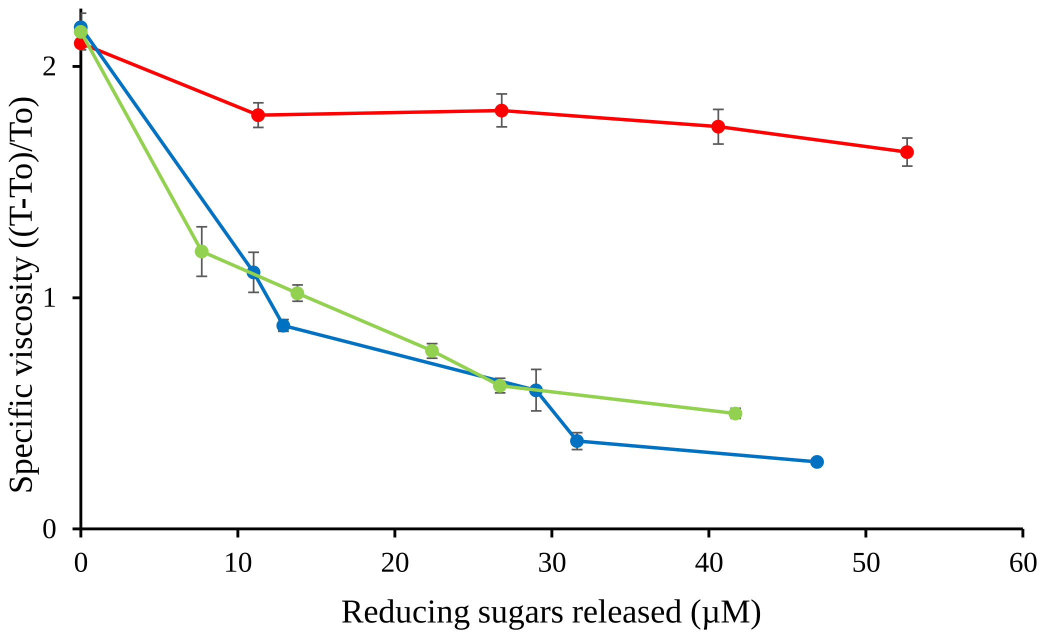

Supplementary Figure S4: HPAEC-PAD analysis of the action of the β-glucosidase Glu3A on the end products generated by a) Cel44O and b) Cel9X on xyloglucan.

Xyloglucan (3.5 g/L) was incubated for 24 h at 37°C with 0.5 µM of Cel44O (**a**) or 0.5 µM of Cel9X (**b**), prior heating at 100 °C for 5 min. The xyloglucan oligosaccharides obtained in each case (black lines, **a** and **b**) were then incubated with 1 µM of Glu3A for 30 min (red lines, **a** and **b**) or 2 h (blue lines, **a** and **b**). The obtained sugars are indicated on top of each peak.

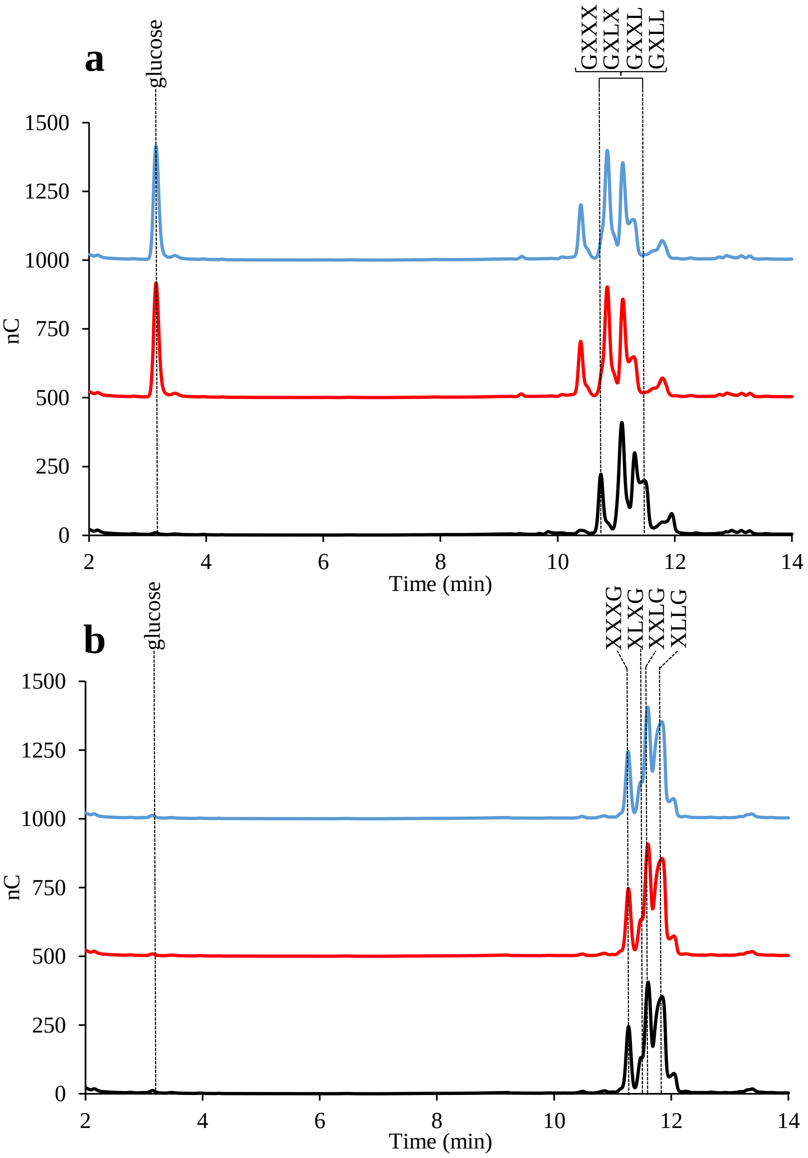

Figure S5: Lack of interaction between the SBP_{2458} and isoprimeverose observed by ITC. The upper part of each panel shows the raw binding heats and the lower part shows the integrated binding heats minus the dilution control heats. The SBP was at 40 µM and the ligand at 4 mM. Similar ITC data were obtained for cellotriose, cellotetraose, glucose, xylose and galactose.

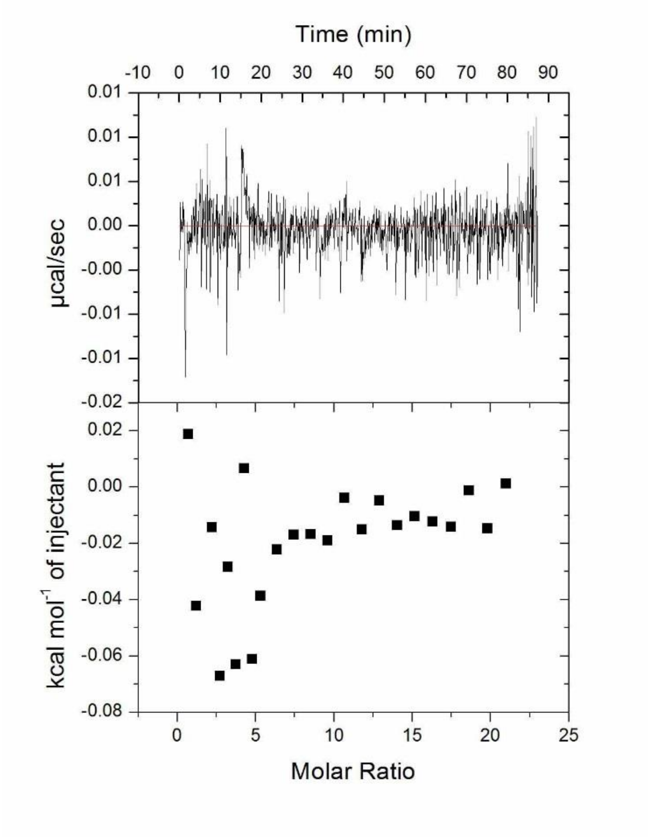

Figure S6: Growth of wild-type and mutant MTL2458 strains of *R. cellulolyticum* on minimal medium supplemented with a) cellobiose (2 g/L) or b) tamarind xyloglucan (3.5 g/L).

The cultures were inoculated (1/33) with cellobiose (2 g/L) grown precultures. The data show the mean of three independent experiments for wild type (black squares) and MTL2458 (open squares) strains, respectively. Bars represent the standard deviations.

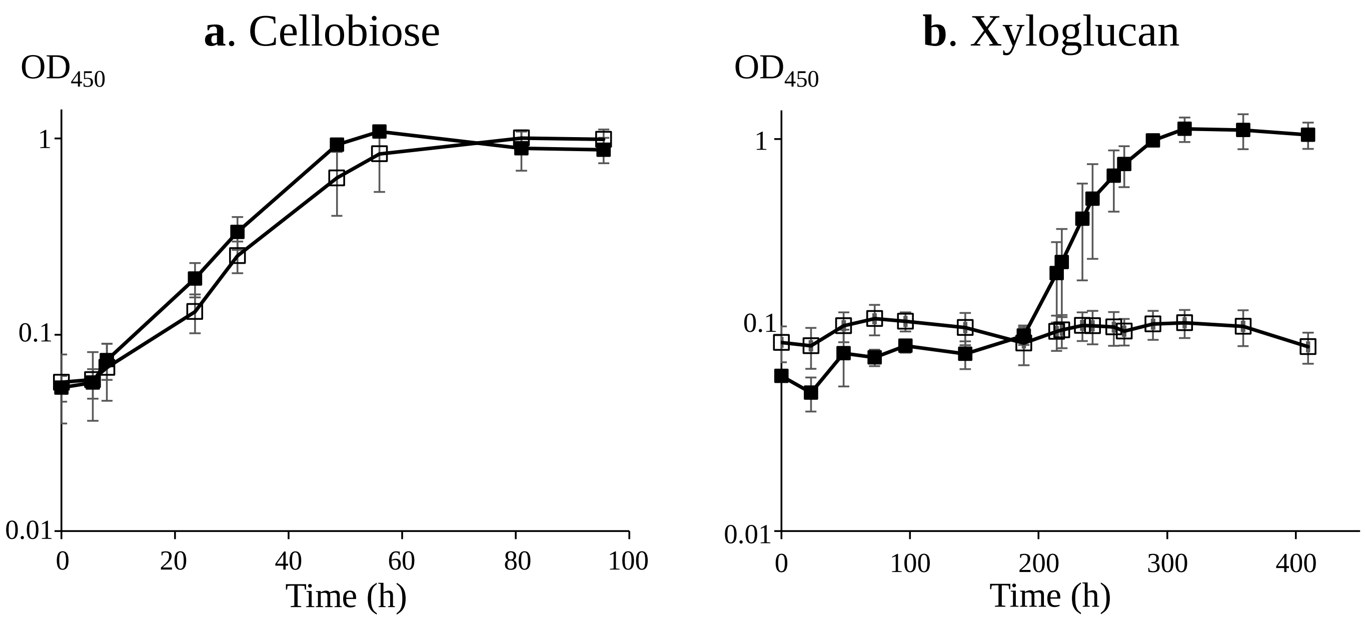

Supplement: Supplementary Information [file srep22770-s1.pdf]
